# Simplified Analysis of Measurement Data from A Rapid *E. coli* qPCR Method (EPA Draft Method C) Using A Standardized Excel Workbook

**DOI:** 10.3390/w12030775

**Published:** 2020-03-11

**Authors:** Molly J. Lane, James N. McNair, Richard R. Rediske, Shannon Briggs, Mano Sivaganesan, Richard Haugland

**Affiliations:** 1 Annis Water Resources Institute, Grand Valley State University, Muskegon, MI 49401, USA; 2 Michigan Department of Environment, Great Lakes, and Energy (EGLE), 525 W. Allegan St., Lansing, MI 48909, USA; 3 Center for Environmental Measurement and Modeling, Office of Research and Development, U.S. EPA, Cincinnati, OH 45268, USA

**Keywords:** *E. coli*, recreational water quality, methods development, qPCR, standard curve model

## Abstract

Draft method C is a standardized method for quantifying *E. coli* densities in recreational waters using quantitative polymerase chain reaction (qPCR). The method includes a Microsoft Excel workbook that automatically screens for poor-quality data using a set of previously proposed acceptance criteria, generates weighted linear regression (WLR) composite standard curves, and calculates *E. coli* target gene copies in test samples. We compared standard curve parameter values and test sample results calculated with the WLR model to those from a Bayesian master standard curve (MSC) model using data from a previous multi-lab study. The two models’ mean intercept and slope estimates from twenty labs’ standard curves were within each other’s 95% credible or confidence intervals for all labs. *E. coli* gene copy estimates of six water samples analyzed by eight labs were highly overlapping among labs when quantified with the WLR and MSC models. Finally, we compared multiple labs’ 2016–2018 composite curves, comprised of data from individual curves where acceptance criteria were not used, to their corresponding composite curves with passing acceptance criteria. Composite curves developed from passing individual curves had intercept and slope 95% confidence intervals that were often narrower than without screening and an analysis of covariance test was passed more often. The Excel workbook WLR calculation and acceptance criteria will help laboratories implement draft method C for recreational water analysis in an efficient, cost-effective, and reliable manner.

## 1. Introduction

Quantitative real-time polymerase chain reaction (qPCR) has become a valuable tool for scientific research due to its specificity, sensitivity, and analysis speed. The technique has been widely implemented in environmental science and public health fields, where it is currently under regulatory consideration as a means for the rapid testing of fecal indicator bacteria in recreational water samples. Because contact with fecal contaminated water increases the likelihood of developing gastrointestinal (GI) illnesses [1,2], public beaches are routinely monitored for the presence of *Enterococcus* or *Escherichia coli* (*E. coli*) to alert beach managers and recreators to incidences of fecal contamination, thereby reducing the risk of recreational exposure [3,4]. Furthermore, current United States Environmental Protection Agency (U.S. EPA) guidelines for microbial recreational water quality are based on the risks of contracting GI illness associated with different levels of these organisms or a specific sequence fragment of their deoxyribonucleic acid (DNA) [4].

At present, there are no epidemiological studies directly demonstrating a relationship between *E. coli* measurements from a qPCR method and swimming related illnesses. Previous studies have demonstrated, however, that results from an *E. coli* qPCR method and an approved *E. coli* culture method, where a health relationship has been established, can show a high degree of correlation and lead to similar recreational beach management decisions [5]. Additional studies have been conducted in 2016–2018 in the state of Michigan to similarly assess the relationships between *E. coli* culture methods and an *E. coli* qPCR method (draft method C) developed by the U.S. EPA (S. Briggs and R. Haugland, personal communications). Any water quality testing method that is intended to be applied across many localities must be robust to the varying environmental conditions intrinsic to water bodies (i.e., turbidity and organic content), easily performed by laboratory analysts, and shown to produce reliable, consistent, and accurate results. Draft method C is being developed by the U.S. EPA as a standardized qPCR method for the quantification of *E. coli* that would provide same-day microbial water quality results. Quantification by qPCR is achieved by fitting a standard curve, also called a calibration curve, to data consisting of the base-10 logarithms (log_10_) of a series of known quantities of target DNA sequences (the standards) and their corresponding measured threshold cycles (Ct values) [6,7]. The fitted standard curve is then used to estimate target gene copy quantities in recreational water samples based on Ct measurements from DNA extracts of these samples.

Early versions of draft method C used a hierarchical Bayesian standard curve model to generate a master standard curve (MSC) [8]. While useful for accurately accounting for the uncertainty in *E. coli* estimates, such determinations of uncertainty are currently not required for routine beach monitoring and the Bayesian MSC model requires specialized statistical software and expertise that could make it impractical for use by labs with limited statistical capabilities. Consequently, a more user-friendly standard curve model was pursued.

The current version of draft method C, being implemented in a Microsoft Excel workbook provided in the supplemental material, uses a classical weighted linear regression (WLR) standard curve model. The draft method C Excel workbook is designed to generate a composite standard curve from the pooled Ct measurement data from a minimum of four independent individual standard curves. The Excel workbook then uses the composite standard curve to automatically quantify mean *E. coli* target gene copies in test sample DNA extracts, e.g., recreational water samples. Another feature of the current version of draft method C that was not used in the previous Bayesian MSC model is a set of quality control acceptance criteria proposed by Sivaganesan et al., [9]. The workbook requires criteria to be met for the y-intercept (hereafter referred to as intercept) and slope of both individual and composite standard curves, as well as for positive and negative controls that are included when analyzing both standard curve and test sample plates. The Excel workbook also uses an analysis of covariance (ANCOVA) model to evaluate intercept and slope values of individual standard curves that meet the acceptance criteria to determine if any large deviations are present. The use of quality assurance and quality control procedures, including the standard curve acceptance criteria, should help to ensure that data quality is maintained [9,10].

To increase U.S. EPA and end user confidence in the current version of draft method C, it was considered important to demonstrate the comparability of the WLR model used in the Excel workbook to the previously used Bayesian MSC model. Consequently, the first goal of this study was to determine whether the WLR model yielded results that were comparable to those of the MSC model. This question was first addressed by determining the similarity of mean composite standard curve intercept and slope estimates obtained from the WLR and MSC models using the Ct values from the DNA standards from a large multi-lab study conducted in 2016 [9]. Furthermore, the variability of draft method C results was previously defined from quantified test sample estimates obtained using the Bayesian MSC model in a separate 2016 study [11]. Thus, we further addressed the question by determining the similarity of *E. coli* target gene copy estimates using MSC standard curves from the 2016 multi-lab study [9] and WLR composite curves generated by the same labs in subsequent studies, together with the same test sample measurement data from 2016 [11] for both. A second goal of this study was to evaluate the impact of implementing the intercept and slope acceptance criteria on the variability and reliability of WLR composite curves generated by multiple labs using the Excel workbook during ongoing recreational water sample analyses in 2016–2018 with draft method C. These evaluations were performed by comparing the variability of WLR standard curve intercept and slope estimates and the ability of the contributing individual curves to pass the ANCOVA test before and after screening individual standard curve data with the proposed acceptance criteria. The frequencies at which the individual standard curves passed or failed also were used as a gauge of lab performance and for troubleshooting logistical and technical problems with standards analyses during the ongoing 2016–2018 studies.

## 2. Materials and Methods

### 2.1. Participants

The present study includes three assessments, each of which employed data from labs that volunteered to participate. Comparison of the WLR and MSC models used data from the 2016 study [9], which included a diverse group of twenty-one labs from across the midwestern and southeastern United States (Table 1). Data for comparing test sample estimates and the impact of acceptance criteria came from a subset of labs in the State of Michigan, which is the only state that has moved forward thus far with draft method C in practice. Moreover, the decision to include labs for the test sample analysis comparisons was based on the completeness of their accepted data sets from the 2016 study [11] and whether ongoing WLR composite curves were available from these labs. Data from eight labs were used for the test sample analysis comparisons and data from eleven labs were used for determining the impact of acceptance criteria analysis. Government, university and county health department labs were represented in all three assessments. Each lab was assigned a unique numeric code (1–21) to maintain anonymity when presenting results.

### 2.2. qPCR Analyses

The qPCR assays (EC23S857 *E. coli* [12] and the Sketa22 (salmon DNA sample processing control)), methods of DNA extractions, and qPCR analysis methods used for all parts of this study are described by Sivaganesan et al., [9] and Aw et al., [11]. Methods used to construct the Bayesian MSCs are described in detail by Sivaganesan et al., [9] and methods used to construct the WLR standard curves are described below. The standards used to generate the WLR and Bayesian MSC standard curve models were prepared and quantified at the U.S. EPA Cincinnati laboratory, as described by Sivaganesan et al., [13]. Participating labs received five standards (where standard number 5 was the lowest concentration) that were shipped overnight on dry ice from a central lab. A sixth standard was prepared on site by most of the labs by 1:1 (v:v) dilution of standard 5 with AE buffer. Each year, all labs used a single lot of TaqMan™ Environmental MM 2.0 (Thermo Fisher Scientific, Waltham, MA, USA) that was confirmed to have little or no underlying *E. coli* DNA contamination for standard curve and test sample analysis.

### 2.3. Standard Curves in the Draft Method C Excel Workbook

Upon completion of instrument analysis of the standards, data were exported and the resulting Ct values copied into the draft method C Excel workbook where the WLR model was fitted. The WLR model has the form

(1) $$Y_{ijk}=\alpha_{i}+{\text{β}}_{i}\log_{10}\left(X_{j}\right)+\varepsilon_{ijk}$$

where *Y*_*ijk*_ is the observed Ct value for replicate *k* of standard concentration *j* in run *i*; α_*i*_ and β_*i*_ are the intercept and slope, respectively, for run *i*; *X*_*j*_ is the known copy number (standard concentration) in standard *j*, and ε_*ijk*_ is the statistical error in the observed threshold cycle. Because variation among observed Ct values in replicates of each standard is approximately inversely proportional to the logarithm of the copy number, the model was fitted to data for each run *i* by weighted least-squares linear regression to stabilize the variance, with multiplicative weight *w*_*j*_ = log_10_(*X*_*j*_) applied to squared errors for each standard *j*. A separate WLR was fitted to Ct values from each individual standard curve, and the externally Studentized residuals [14] (p. 20) were examined to identify and remove up to two outliers from the triplicate analyses of standards from each individual standard curve’s data set if needed. The WLR model was then re-fitted to the retained data for each analysis, and the intercept and slope values for each individual standard curve were assessed for acceptability based on the proposed standard curve acceptance criteria developed for draft method C (36.66 to 39.25 for intercept, −3.23 to −3.74 for slope) [9]. The Excel workbook also screens for other quality control parameters such as the positive control (calibrator) Sketa22 (18.58 to 22.01) and *E. Coli* assay (26.48 to 29.63) Ct values; and negative controls such as no-template control (NTC), un-spiked filter (filter blank), and lower limit of quantification (LLOQ) Ct values, which are determined directly from each composite standard curve and not the global value proposed in Sivaganesan et al., [9]. The LLOQ was the Ct value of the estimated 95% confidence interval (CI) upper bound at the lowest concentration. If an individual standard curve met these criteria it was considered ‘passing’; if a curve failed one or more of the criteria, it was considered ‘failing’. The intercept and slope data for each passing standard curve from each lab were then assessed by the workbook with an ANCOVA model to determine if there was strong evidence (*α* = 0.01) that any parameter estimates differed among runs. If there was no strong evidence of a difference (*p* > 0.01 for both parameters), results from the individual curves were pooled, and a WLR model was fitted to estimate the composite curve intercept, slope, and 95% CIs for each lab. The current version of the draft method C workbook requires a minimum of four passing individual curves to generate the composite curve used to analyze recreational water samples. However, for the purposes of our study, in six instances, four of which were from the 6-point standard curves (Supplementary Table S1), the minimum of four individual standard curves was relaxed to three to include data from all actively participating Michigan network labs.

### 2.4. Data Analysis

#### 2.4.1. WLR and Bayesian MSC Standard Curve Model Comparisons

In 2016, twenty-one labs (Table 1, column 3) analyzed four or five individual standard curves for a total of ninety-one curves analyzed. Mean intercept and slope estimates from the Bayesian MSC model for individual labs were taken from Sivaganesan et al., [9]; the 95% Bayesian Credible Intervals (BCIs) are provided in the supplemental material (Supplementary Table S2). WLR composite curve intercept and slope estimates were determined as described in Section 2.3. The ANCOVA evaluation and standard curve acceptance criteria were applied to the WLR model only (not the Bayesian MSC model) using the Excel workbook. Mean intercept and slope estimates, and corresponding 95% BCIs and CIs, from the Bayesian MSC and WLR models, respectively, were plotted using R software (V 3.6.1, Vienna, Austria) for a visual comparison of each lab’s results.

#### 2.4.2. Comparison of Test Sample *E. coli* Estimates by the Two Models

Data from eight labs were used for this assessment. Ct measurement data from one filter each, analyzed in duplicate, of a six-sample (Table 2) subset of the eighteen water samples described in Aw et al., [11] and corresponding positive control (calibrator) Ct data from three filters (analyzed in duplicate producing six measurements) from the 2016 study were chosen for further analysis in our study. In each case, data from only the first of three replicate test sample filters in the 2016 study were selected in accordance with the common practice of analyzing only one filter per water sample for beach monitoring in Michigan. The test sample data represented nearly the entire range of *E. coli* concentrations examined in the 2016 study. Furthermore, test sample data were from labs that met all draft method C quality control criteria for these samples and had ongoing 2016–2018 WLR composite curves available for comparison. *E. coli* log_10_ target gene copy estimates were determined using the mean standard curve intercept and slope values from the 2016 Bayesian MSC model and mean values from one corresponding ongoing WLR composite curve (nearest in time to the 2016 study) from each lab. *E. coli* target gene copy estimates were determined, as per the draft method C Excel workbook, from the intercept and slope values and from the mean positive control (calibrator) and test sample Ct values using the following formula:

(2) $$U_{\text{smp}}=\left(Y_{\text{smp}}-\left(S_{\text{smp}}-S_{\text{cal}}\right)-\alpha_{\text{std}}\right)/{\text{β}}_{\text{std}}$$

where *U*_smp_ is the estimated mean sample log10 copy number of the target gene, *Y*_smp_ is the mean sample *E. coli* Ct, *S*_smp_ is the mean sample Sketa22 Ct, *S*_cal_ is the mean calibrator Sketa22 Ct, α_std_ is the estimated intercept for the standard curve, and β_std_ is the estimated slope of the standard curve. This formula adjusts the sample *E. coli* Ct value (*Y*_smp_) to remove any anomalous changes due to interference or facilitation by the test sample matrix, as indicated by the difference between the sample and positive control (calibrator) Sketa22 Ct values (│*S*_smp_ − *S*_cal_│ > 0), then uses this adjusted Ct value in the inverted WLR regression equation to determine the corresponding log_10_ copy number of the target gene in the sample (*U*_smp_). Mean *E. coli* target gene copy concentrations from the two models were then plotted side-by-side using R Software (V3.6.1) and visually compared. Individual standard curve acceptance criteria and the ANCOVA evaluation were assessed in the draft method C Excel workbook for the WLR composite standard curves only.

#### 2.4.3. Impact of Acceptance Criteria on WLR Intercept and Slope Estimates

Data from eleven labs were used for this assessment. For ongoing lab composite standard curve development in 2016–2018, the acceptance criteria were automatically applied to the individual standard curves by the draft method C Excel workbook. Acceptance criteria included ranges for intercept (36.66 to 39.25) and slope (−3.23 to −3.74). Additionally, individual passing curves had to pass an ANCOVA analysis (*α* = 0.01) as implemented with the Excel workbook. Curves were generated using data from five standards as described in Sivaganesan et al., [9] however, an additional sixth standard was also prepared (described in section 2.2) and analyzed by most labs to extend the method’s range of quantification. Twenty-one 5-point (using five standards) and nineteen 6-point (using 6 standards) composite curves were created between 2016 and 2018 and were evaluated for this portion of the study. The number of individual standard curves analyzed by the different labs each year varied from four to ten (Table S1). Individual standard curves were used to generate composite 5-point and 6-point standard curve in two ways: 1) using data from all individual curves without acceptance criteria screening and 2) using only passing individual curves after acceptance criteria screening was performed in the workbook. Thus, the number of individual standard curves used to create the composite curves varied. WLR 5-point and 6-point composite standard curve mean intercept and slope parameters and 95% CI intervals from the two methods described above were compiled and plotted separately using R software (V3.6.1) to visually assess the impact of the proposed acceptance criteria.

## 3. Results

### 3.1. Bayesian MSC and WLR Standard Curve Model Comparisons

The goal of these comparisons was to assess whether results from the simpler and more user-friendly WLR model approximate those from the MSC model and thus can provide a suitable replacement. All passing individual standard curves analyzed by the twenty-one labs in 2016 had intercept and slope estimates that passed the ANCOVA test allowing a WLR composite standard curve to be produced for every lab. However, lab code 21 was excluded from the model comparisons since it was not used in the Bayesian MSC analysis [9].

**Figure 1.**
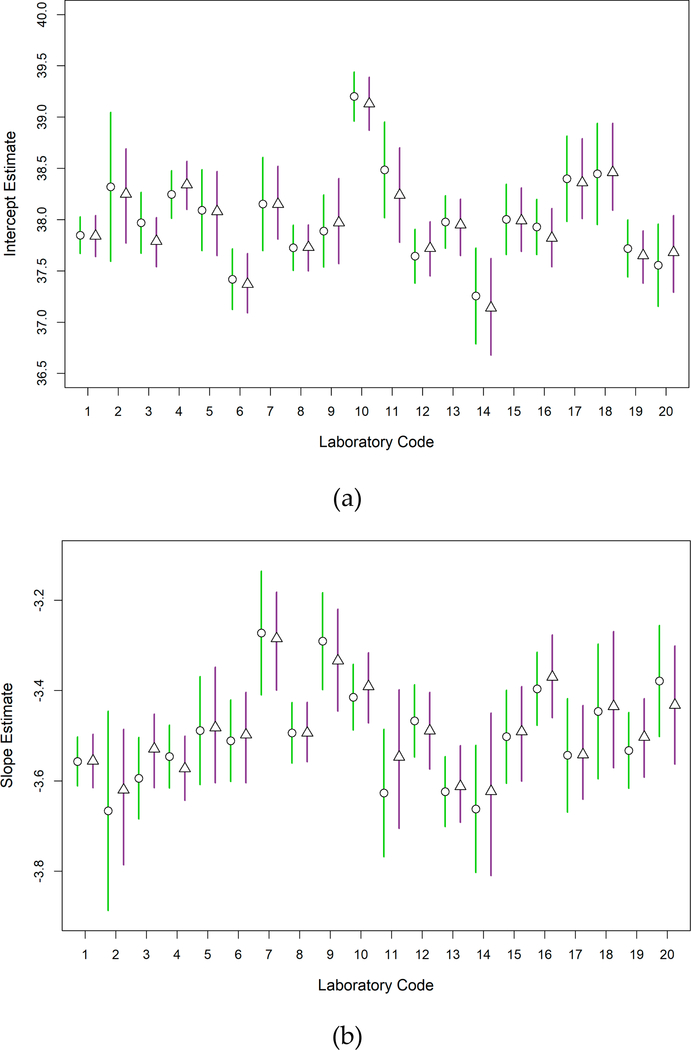
Weighted Linear Regression (WLR) and Master Standard Curve (MSC) mean intercept and slope estimates. Comparison of (**a**) mean intercept and (**b**) slope estimates of the 2016 standard curve composite WLR (green bar with open circle) and Bayesian MSC (purple bar with open triangles) models. Open circles and triangles on bars indicate the mean least-squares and Bayesian intercept and slope estimates, respectively. Vertical lines represent the WLR 95% CI (green) and Bayesian MSC 95% BCI (purple).

Estimates of the mean intercepts and the corresponding 95% CI and BCI calculated from WLR and Bayesian MSC models were similar for each lab (Figure 1a). Furthermore, the mean values that would be used in the standard curves from each model were within the uncertainty ranges of the other model in each case. Similar results were observed for slope values (Figure 1b). Each lab’s mean intercept and slope, and 95% confidence intervals can be found in Supplementary Table S3.

### 3.2. Comparison of Test Sample E. coli Estimates by the Two Models

**Figure 2.**
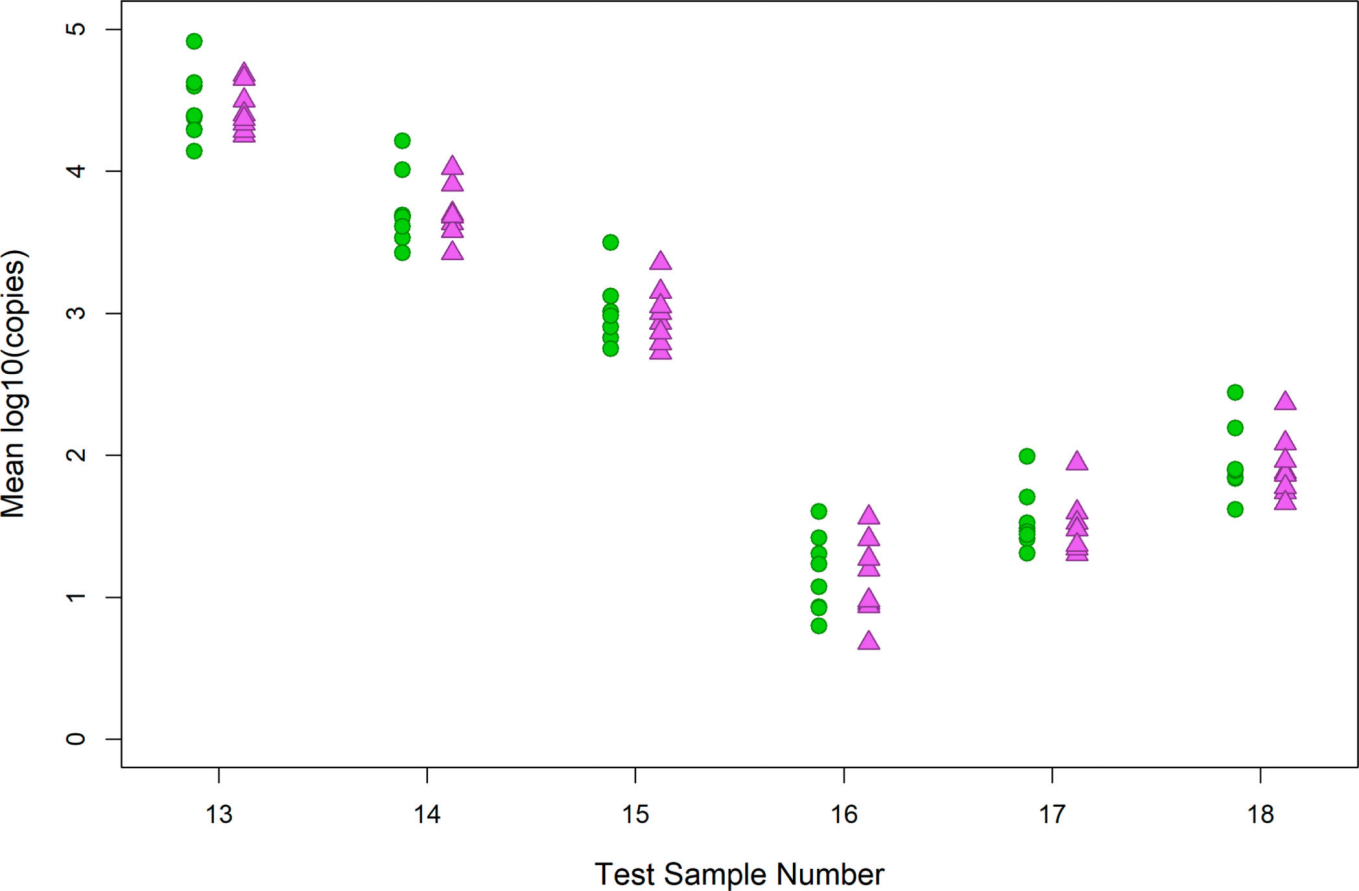
Test sample *E. coli* target gene copy results. Green circles (WLR) and purple triangles (MSC) represent *E. coli* concentrations quantified with the two models. X-axis is the test sample identification number. Y-axis is the mean log_10_ *E. coli* copies per reaction.

The goal of this analysis was to examine the effects of the two models on mean test sample *E. coli* estimates (Supplementary Table S4) and the between-lab variability of these estimates. The mean estimates of *E. coli* log10 target gene copies from the different labs were highly overlapping for all water samples using the standard curve intercept and slope values from the two models (Figure 2).

### 3.3. Impact of Acceptance Criteria on WLR Intercept and Slope Estimates

**Figure 3.**
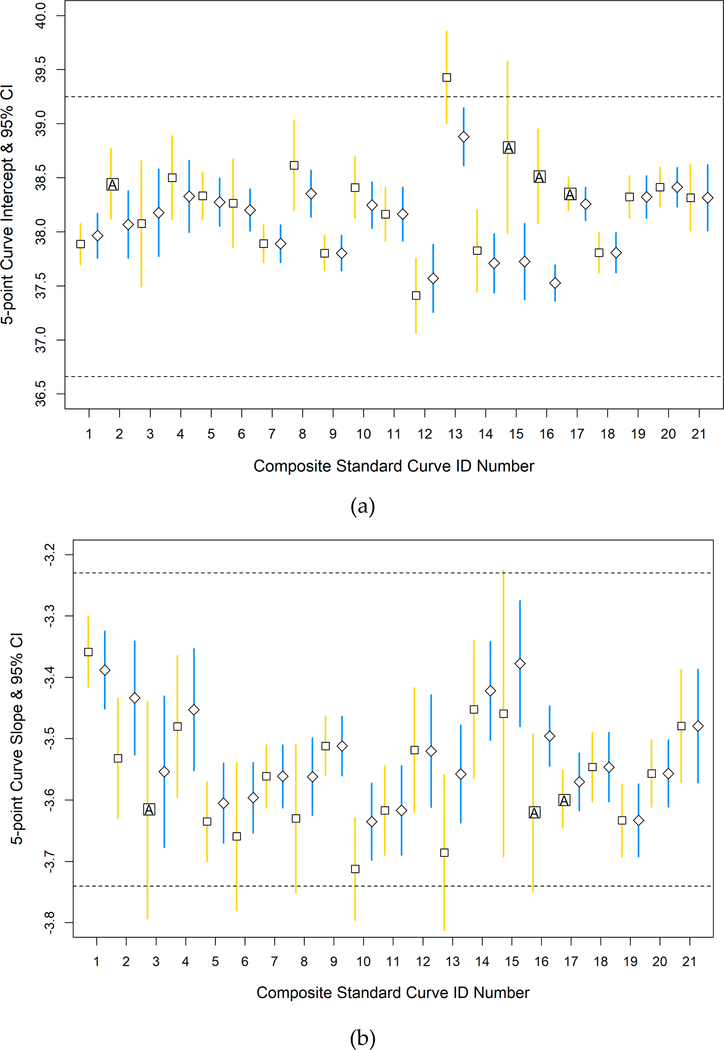
Mean 5-point composite standard curves intercept and slope estimates and 95 % confidence intervals (CIs). (**a**) Mean intercept; and (**b**) mean slope estimates before (open squares with gold lines) and after (open diamonds with blue lines) data screening with the proposed acceptance criteria. Vertical lines represent 95% CIs. Horizontal dashed lines represent the standard curve acceptance criteria range. Boxes with an inset “A” show individual standard curves without acceptance criteria enforced that did not pass the analysis of covariance (ANCOVA) therefore a composite curve value was not calculated in the Excel workbook.

For ongoing sample analyses in 2016–2018, each individual curve’s intercept was required to meet the intercept and slope acceptance criteria in addition to passing ANCOVA analysis, as described in Section 2.4.3, before the development of composite curves. The impact of imposing the acceptance criteria, including the ANCOVA, on five-point individual standard curves was examined by visually assessing the variability in composite standard curve mean intercept and slope values before and after screening using the above criteria. When acceptance criteria were not imposed, there were four instances of intercept and three instances of slope values where the individual curves did not pass the ANCOVA (Figure 3a and b). In contrast, when only passing individual standard curves were analyzed, there were no instances of a failing ANCOVA. In one instance, the five-point composite standard curve mean intercept value fell outside the global acceptance range (standard curve 13; Figure 3a) when individual standard curves were used without acceptance criteria screening but was within the global acceptance range when individual curves were screened. No composite curve intercept or slope 95 % CI from individual standard curves passing the acceptance criteria fell outside the global acceptance ranges. The 95 % CIs also were often narrower for composite curve intercept and slope values when generated from passing individual standard curves compared to when the acceptance criteria were not used to screen the curves.

**Figure 4.**
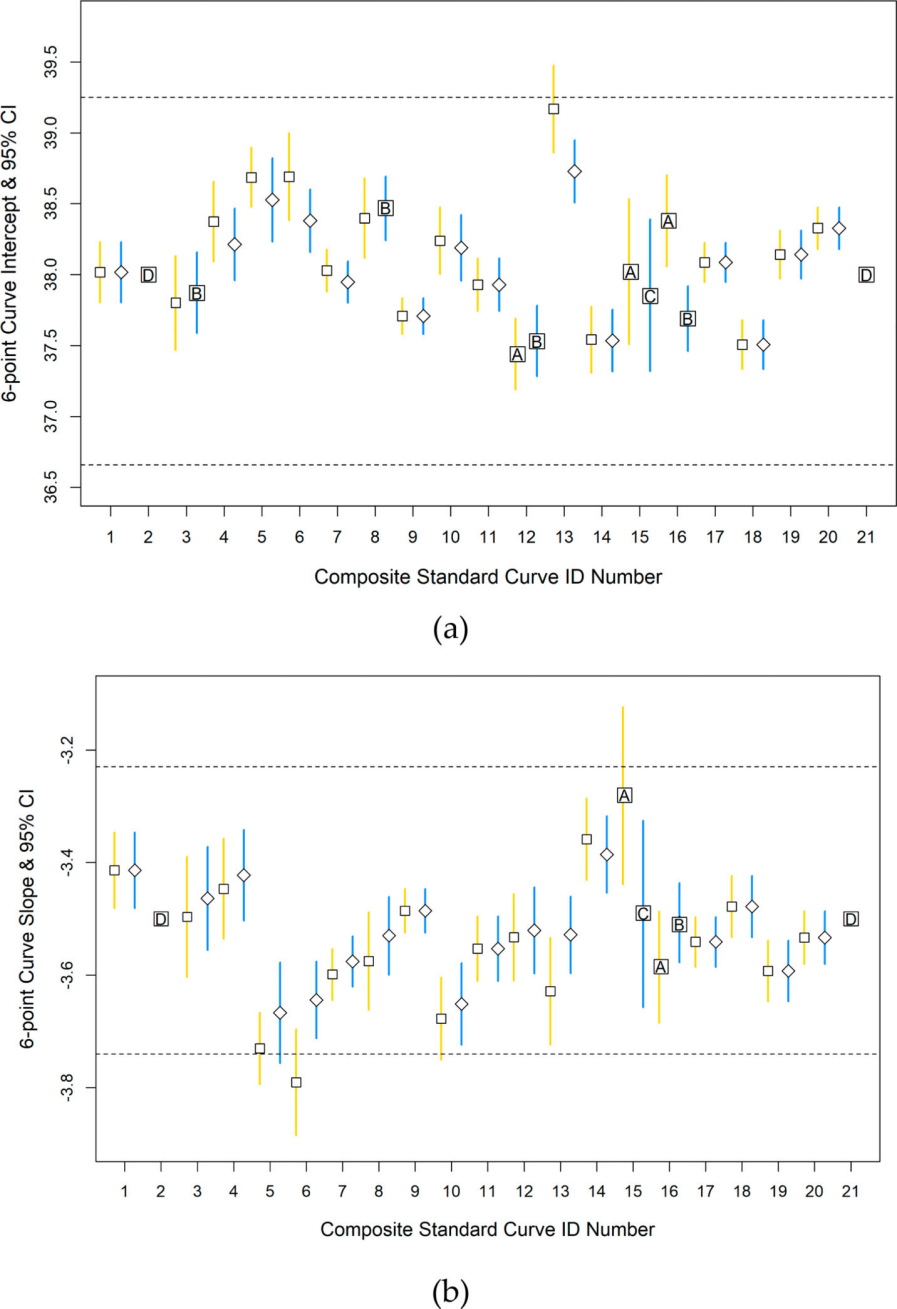
Mean 6-point composite standard curves intercept and slope estimates and 95% (confidence intervals) CIs. (**a**) Mean intercept; and (**b**) Mean slope estimates before data screening (open squares with gold lines) and after (open diamonds with blue lines). Vertical lines represent 95% CIs. Horizontal dashed lines represent the standard curve acceptance criteria range. Boxes with an inset ‘A’ show individual standard curves without acceptance criteria enforced that did not pass the ANCOVA therefore a composite curve value would not be calculated; the box with an inset ‘B’ shows individual standard curves that were screened with acceptance criteria and failed the analysis of covariance (ANCOVA); the box with an inset ‘C’ indicates an insufficient number of passing individual standard curves to generate a composite curve; and boxes with an inset ‘D’, no data was collected.

Individual and composite standard curve intercept and slope estimates using the sixth standard with a lower quantity of target sequences (six-point curves) were also evaluated during ongoing analyses in 2016–2018. Results were not available for curves 2 and 21 because the sixth standard was not analyzed by that lab; and for curve 15 because there were insufficient passing individual standard curves to perform the ANCOVA analysis (Figure 4). Three intercept and two slope values of composite curves generated from unscreened individual standard curves did not pass the ANCOVA. Composite curve intercept values from individual curves that passed the screening failed the ANCOVA four times and the slope value failed once. In all instances except one (Figure 4b, curve 5), the composite curve mean intercept and slope values and their 95% CIs fell within the global acceptance range of the proposed criteria when the individual standard curves were screened and, as was observed for the five-point curve results, the 95% CI ranges were often narrower for composite curves generated from only acceptable individual standard curves. The mean intercept and slope, and their lower and upper bounds of the five-point and six-point composite curves, before and after data were screened with acceptance criteria are presented in Supplementary Table S5.

## 4. Discussion and Conclusions

### 4.1. WLR and Bayesian MSC Standard Curve Model Comparisons

This assessment compared two standard curve models: a simplified WLR model that is currently being used in a draft method C Excel workbook; and the Bayesian MSC model that was previously used to define the variability of draft method C results in a large multi-lab study [11]. While different approaches were used to determine the uncertainty ranges of the intercept and slope estimates from the two models: 95% CI for WLR; and 95% BCI for MSC, the magnitude of these ranges was the same or similar in most instances and the ranges were highly overlapping for all labs (Figure 1, Supplementary Table S4). Consistent with these observations, the mean intercept and slope estimates from the two models were also highly similar for each of the labs. Given that only the mean intercept and slope estimates are used for the quantification of test samples in draft method C, it is significant that the mean values from each model were always within the uncertainty ranges of the other model for each of the labs. These results suggest that *E. coli* target gene copies in test samples that are based on standard curves generated by the WLR and MSC models will not show meaningful differences, thus providing preliminary evidence to support the use of the WLR model as an adequate approximation of the MSC model for standard curve development.

### 4.2. Comparison of Test Sample E. coli Estimates by the Two Models

Estimates of *E. coli* target gene copies from standard curve-based qPCR methods are dependent on both the intercept and slope variables. Therefore, our independent comparisons of the intercept and slope estimates from the WLR and MSC models do not fully predict the similarity of test sample results that the two models would produce, nor do they reflect the performance of the workbook’s WLR model in ongoing studies by the labs. To further address these questions, mean intercept and slope values determined from the MSC model in the 2016 study and corresponding, selected WLR values generated by the same labs from analyses of the same standards in subsequent studies in 2016–2018 were used to determine estimates of target gene copies in test samples from the 2016 study using the same test sample measurement data from the 2016 study in each case. Because the target gene copy estimates obtained from the MSC and WLR standard curves incorporated the differences in both intercept and slope values from the two models and used ongoing 2016–2018 WLR curves for each lab, the highly overlapping results among the different labs for each sample provided further support for adopting the WLR model in the workbook for ongoing standard curve development.

### 4.3. Impact of Acceptance Criteria on WLR Intercept and Slope Estimates

Quantification of *E. coli* by qPCR requires a standard curve model and variation and error can be introduced during each step of the analysis [15]. Factors such as storage time, storage temperature of standards and reagents, precision of dilutions, and pipette calibration can influence the accuracy of standard concentration measurements used to create the standard curve [8,16]. Additionally, differences in equipment used within and between laboratories as well as the ability of analysts to pipette uniformly contribute to variations in the individual standard curves. One way to reduce variation is by imposing quality control procedures, such as standard curve acceptance criteria, to help ensure results generated by different labs are maintained within a defined range.

When individual curves were not screened, the mean intercept and slope values of the composite curves still fell within the acceptance ranges in most cases, however, the 95% CIs frequently extended outside. The mean intercept and slope values of the 5-point composite curves as well as their 95% CIs consistently fell within the proposed acceptance ranges when individual curves from ongoing studies in 2016–2018 were screened. Furthermore, 95% CIs were equal or narrower for most five-point composite standard curves after screening individual curves. The narrower intervals generated after screening suggest less uncertainty in the mean parameter values.

ANCOVA analysis also was performed on the individual curves comprising each five- or six-point composite curve in the workbook. Intercept and slope values of the individual standard curves cannot be significantly different for the Method C workbook to generate composite curves. Several of the five-point composite curves failed the ANCOVA analysis when the individual curves were not screened, however, all five-point composite curves passed when the individual curves were screened with the intercept and slope acceptance criteria. In contrast, several six-point composite curves failed ANCOVA analyses even after screening individual curves with the acceptance criteria. Six-point standard curves have been successfully used to extend the draft method C quantification range for developing relationships between qPCR and culture data (R. Haugland, personal communication) but their use is not presently considered to be essential for routine beach monitoring. The increased ANCOVA failure rate of six-point curves among the labs after screening could be related to the decentralized preparation of the sixth standard by each lab and thus may highlight previous recommendations that reference DNA be verified and provided by a single supplier [9,17].

### 4.4. Draft Method C Implementation

Although the MSC model can provide a better understanding of the sources of uncertainty in these estimates [8], the results of this study suggest that the use of the WLR model will have little effect on the determination of mean estimates of *E. coli* target gene copies in test samples in draft method C. As such, the WLR model incorporated into the Excel workbook can be expected to provide a reliable but simpler alternative to the MSC model for recreational water testing by draft method C. Our results further suggest that the automatic incorporation of individual and composite standard curve acceptance criteria in the workbook may reduce the uncertainty of quantifying *E. coli* target gene copy estimates in some instances and at least should provide greater consistency in the results. Largely due to resource limitations by many end-user labs, characterizing the uncertainty of test sample estimates is not presently a priority in draft method C implementation for beach monitoring. However, it could be important for other applications of the method and this issue has been addressed in recent U.S. EPA microbial source tracking (MST) methods [10].

Use of either a composite WLR or a Bayesian MSC model in place of individual standard curves on each reaction plate, which is typical of many qPCR methods [10,18,19], increases sample analysis efficiency, reduces the costs of preparing or purchasing standards and increases the number of test samples that can be analyzed on each plate. A previous report [16] suggested that the use of master (or composite) curves may be most efficient for studies involving large numbers of continuous sample analyses over time, which may be typical for beach monitoring programs, while generating standard curves on each reaction plate may be more suitable for smaller-scale or more sporadic analyses which could be the case for many MST studies. However, it is also recognized that care must be taken when applying master or composite standard curves to test sample data from multiple plate runs. This issue is partially addressed in draft method C by the analyses of positive control samples on each plate. Acceptance criteria for these analysis results were also established in the 2016 multi-lab study [9,11] and are incorporated into the draft method C workbook. Beyond this, guidance also has been established for labs implementing draft method C wherein the minimum quantity of four acceptable individual standard curves, used for composite curve development, should be obtained from a maximum of six independent curve runs. With the incorporation of some flexibility into this guidance, e.g., curves where known or suspected technical or logistical issues have been identified can be ignored, all the labs performing ongoing recreational water sample analyses in 2016–2018 using draft method C (Table 1) were able to meet this basic minimum requirement with their five-point curves.

Our findings support the use of draft method C with the Excel workbook and proposed acceptance criteria as a rapid, standardized protocol for estimating *E. coli* densities in recreational waters. The Excel workbook, WLR calculation, and acceptance criteria will help public laboratories and scientists perform draft method C in an efficient and cost-effective manner that produces reliable data.

## Supplementary Material

Supplement1 Table S1: Number of standard curves analyzed by each lab, Table S2: Intercept and slope 95% Bayesian MSC Credible Intervals, Table S3: Mean intercept and slope values from Weighted Linear Regression (WLR) by lab, Table S4: Test sample *E. coli* estimates, Table S5: Mean intercept and slope before and after data screening; Microsoft Excel Workbook: Method C Workbook.

## Figures and Tables

**Table 1.** Participating labs. List of labs participating in the three assessments conducted in the present study. An ‘x’ indicates that data from the respective lab (row) were used in the respective assessment (column). (WLR = Weighted Linear Regression; MSC = Master Standard Curve).

Laboratory	Location	WLR vs MSC	Test Sample Analysis	Acceptance Criteria Impact (2016–2018)

Central Michigan District Health Dept., Assurance Water Laboratory	Gladwin, MI , 48624, USA	x		x
City of Racine Public Health Dept.	Racine, WI, 53403, USA	x		
Ferris State University, Shimadzu Core Laboratory	Big Rapids, MI, 49307, USA	x	x	x
Georgia Southern University, Dept. of Environmental Health Sciences	Statesboro, GA, 30458, USA	x		
Grand Valley State University, Annis Water Resources Institute	Muskegon, MI, 49441, USA	x	x	x
Health Dept. of Northwest Michigan, Northern Michigan Regional Laboratory	Gaylord, MI, 49735, USA	x		
Kalamazoo County Health and Community Services Laboratory	Kalamazoo, MI, 49001, USA	x	x	x
Lake Superior State University, Environmental Analysis Laboratory	Sault St. Marie, MI, 49783, USA	x	x	x
Marquette Area Wastewater Facility	Marquette, MI, 49855, USA	x		x
Michigan State University, Department of Fisheries and Wildlife	East Lansing, MI, 48824, USA	x		
Northeast Ohio Regional Sewer District, Environmental and Maintenance Services Center	Cuyahoga Heights, OH, 44125, USA	x		
Oakland County Health Division Laboratory	Pontiac, MI, 48341, USA	x	x	x
Oakland University, HEART Laboratory	Rochester, MI, 48309, USA	x	x	x
U.S. EPA National Exposure Research Laboratory	Cincinnati, OH, 45268, USA	x	x	x
United States Geological Survey, Upper Midwest Water Science Center	Lansing, MI, 48911, USA	x		x
U.S. National Parks Service, Sleeping Bear Dunes Water Laboratory	Empire, MI, 49360, USA	x		
Saginaw County Health Dept. Laboratory	Saginaw, MI, 48302, USA	x		
Saginaw Valley State University, Dept. of Chemistry	University Center, MI, 48710, USA	x	x	x
University of Illinois at Chicago, School of Public Health	Chicago, IL, 60612, USA	x		
University of North Carolina at Chapel Hill, Institute of Marine Sciences	Morehead City, NC, 28557, USA	x		
University of Wisconsin-Oshkosh, Environmental Research Laboratory	Oshkosh, WI, 54901, USA	x		

**Table 2.** Test sample descriptions. Test sample ID’s, type and concentrations as described in Aw et al., [11].

Test Sample ID	Test Sample Type	Test Sample Concentration (*E. coli*/100 mL)

13	Ambient	86,596
14	Low Dilution	20,535
15	High Dilution	2371
16	Ambient	Not Determined +
17	Low Spike	200 *
18	High Spike	800 *

+ Sample *E. coli* cell concentration not quantified.

* Estimated *E. coli* cell concentration based on spike levels.
