# Supplementary material for: Simplified Analysis of Measurement Data from A Rapid E. coli qPCR Method (EPA Draft Method C) Using A Standardized Excel Workbook
Source: Water (Basel). Author manuscript; Available in PMC 2021 Mar 11. (PMC7252523; doi:10.3390/w12030775)
Supplement: Supplement1 — Table S1: Number of standard curves analyzed by each lab, Table S2: Intercept and slope 95% Bayesian MSC Credible Intervals, Table S3: Mean intercept and slope values from Weighted Linear Regression (WLR) by lab, Table S4: Test sample E. coli estimates, Table S5: Mean intercept and slope before and after data screening; Microsoft Excel Workbook: Method C Workbook. [file NIHMS1579161-supplement-Supplement1.zip › water-733572-final suppl/Lane et al. - Supplementary Materials.docx]

Supplemental Material

**Table S1.** Number of standard curves analyzed by each lab. The total number (num.) of individually analyzed standard curves and the number of individual standard curves used to develop the composite curve, including the year individual standard curves were analyzed.

| **Composite Standard Curve ID** | **Total Num. Individual Curves Analyzed** | **Num. Individual Curves Used for Composite Curve** | **Year Analyzed** |
| --- | --- | --- | --- |
| 5-point Curve |  |  |  |
| 1 | 6 | 5 | 2017 |
| 2 | 6 | 4 | 2017 |
| 3 | 7 | 4 | 2017 |
| 4 | 4 | 3* | 2017 |
| 5 | 6 | 5 | 2018 |
| 6 | 6 | 5 | 2018 |
| 7 | 8 | 8 | 2018 |
| 8 | 8 | 4 | 2018 |
| 9 | 8 | 8 | 2018 |
| 10 | 6 | 4 | 2018 |
| 11 | 6 | 6 | 2018 |
| 12 | 8 | 6 | 2018 |
| 13 | 10 | 6 | 2018 |
| 14 | 6 | 4 | 2018 |
| 15 | 6 | 3* | 2018 |
| 16 | 10 | 5 | 2018 |
| 17 | 10 | 9 | 2016 |
| 18 | 6 | 6 | 2017 |
| 19 | 6 | 6 | 2017 |
| 20 | 7 | 7 | 2018 |
| 21 | 5 | 5 | 2016 |
| 6-point Curve |  |  |  |
| 1 | 6 | 6 | 2017 |
| 2 | 0 | 0 | NA |
| 3 | 7 | 6 | 2017 |
| 4 | 4 | 3* | 2017 |
| 5 | 6 | 3* | 2018 |
| 6 | 6 | 3* | 2018 |
| 7 | 8 | 7 | 2018 |
| 8 | 8 | 6 | 2018 |
| 9 | 8 | 8 | 2018 |
| 10 | 6 | 5 | 2018 |
| 11 | 6 | 6 | 2018 |
| 12 | 8 | 6 | 2018 |
| 13 | 10 | 6 | 2018 |
| 14 | 6 | 5 | 2018 |
| 15 | 6 | 1* | 2018 |
| 16 | 10 | 6 | 2018 |
| 17 | 10 | 10 | 2016 |
| 18 | 6 | 6 | 2017 |
| 19 | 6 | 6 | 2017 |
| 20 | 7 | 7 | 2018 |
| 21 | 0 | 0 | NA |

* Indicates composite curves where the four individual curve requirement was relaxed for this study.

**Table S2.** Intercept and slope 95% Bayesian MSC Credible Intervals. 95% BCIs for intercept and slope parameters as determined from 5-point standard curves analyzed in 2016. LB = Lower Bound; UB = Upper Bound. Bayesian MSC mean intercept and slope values can be found in Sivaganesan et al. [8].

| **Lab Code** | **Intercept** | | **Slope** | |
| --- | --- | --- | --- | --- |
|  | **LB** | **UB** | **LB** | **UB** |
| 1 | 37.64 | 38.04 | −3.615 | −3.497 |
| 2 | 37.77 | 38.69 | −3.786 | −3.486 |
| 3 | 37.54 | 38.02 | −3.615 | −3.452 |
| 4 | 38.10 | 38.57 | −3.643 | −3.501 |
| 5 | 37.65 | 38.47 | −3.604 | −3.348 |
| 6 | 37.09 | 37.67 | −3.604 | −3.404 |
| 7 | 37.81 | 38.52 | −3.399 | −3.182 |
| 8 | 37.50 | 37.95 | −3.558 | −3.426 |
| 9 | 37.57 | 38.40 | −3.446 | −3.220 |
| 10 | 38.87 | 39.39 | −3.472 | −3.316 |
| 11 | 37.78 | 38.70 | −3.705 | −3.398 |
| 12 | 37.45 | 37.98 | −3.574 | −3.404 |
| 13 | 37.65 | 38.20 | −3.692 | −3.522 |
| 14 | 36.68 | 37.62 | −3.810 | −3.450 |
| 15 | 37.69 | 38.31 | −3.601 | −3.391 |
| 16 | 37.54 | 38.11 | −3.460 | −3.277 |
| 17 | 38.01 | 38.79 | −3.641 | −3.433 |
| 18 | 38.09 | 38.94 | −3.571 | −3.269 |
| 19 | 37.38 | 37.89 | −3.592 | −3.418 |
| 20 | 37.29 | 38.04 | −3.563 | −3.301 |

**Table S3.** Mean intercept and slope values from Weighted Linear Regression (WLR) by lab. Each lab’s WLR mean intercept and slope with the 95% Confidence Intervals (CI).

| **Lab ID** | **Mean Intercept**  **(CI)** | **Mean Slope**  **(CI)** |
| --- | --- | --- |
| 1 | 37.84  (37.64–38.04) | −3.56  (−3.61–−3.50) |
| 2 | 38.25  (37.77–38.69) | −3.67  (−3.89–−3.45) |
| 3 | 37.79  (37.54–38.02) | −3.59  (−3.68–−3.50) |
| 4 | 38.34  (38.10–38.57) | −3.55  (−3.62–−3.48) |
| 5 | 38.08  (37.65–38.47) | −3.49  (−3.61–−3.37) |
| 6 | 37.37  (37.09–37.67) | −3.51  (−3.60–−3.42) |
| 7 | 38.15  (37.81–38.52) | −3.27  (−3.41–−3.14) |
| 8 | 37.73  (37.50–37.95) | −3.49  (−3.56–−3.43) |
| 9 | 37.97  (37.57–38.40) | −3.29  (−3.40–−3.18) |
| 10 | 39.13  (38.87–39.39) | −3.41  (−3.49–−3.34) |
| 11 | 38.24  (37.78–38.70) | −3.63  (−3.77–−3.49) |
| 12 | 37.72  (37.45–37.98) | −3.47  (−3.55–−3.39) |
| 13 | 37.95  (37.65–38.20) | −3.62  (−3.70–−3.55) |
| 14 | 37.14  (36.68–37.62) | −3.66  (−3.80–−3.52) |
| 15 | 37.99  (37.69 – 38.31) | −3.50  (−3.61–−3.40) |
| 16 | 37.82  (37.54–38.11) | −3.40  (−3.48–−3.32) |
| 17 | 38.36  (38.01–38.79) | −3.54  (−3.67–−3.42) |
| 18 | 38.46  (38.09–38.94) | −3.45  (−3.60–−3.30) |
| 19 | 37.65  (37.38–37.89) | −3.53  (−3.62–−3.45) |
| 20 | 37.68  (37.29–38.04) | −3.38  (−3.50–−3.26) |

**Table S4.** Test sample *E. coli* estimates. Weighted Linear Regression (WLR) and Bayesian Master Standard Curve (MSC) test sample log_10_ *E. coli* estimate ranges, standard deviations (Std.Dev), and individual lab results. MSC estimates were determined as described in Sivaganesan et al. [8]. WLR estimates were produced by the Draft Method C Excel workbook using a composite standard curve and test sample Ct values.

| **Sample ID** | **WLR Range** | **WLR Std. Dev.** | **MSC Range** | **MSC Std. Dev.** | **Lab ID** | **Lab WLR Result** | **Lab MSC Result** |
| --- | --- | --- | --- | --- | --- | --- | --- |
| 13 | 4.010–4.915 | 0.245 | 3.909–4.680 | 0.162 | 1 | 4.598 | 4.499 |
|  |  |  |  |  | 2 | 4.915 | 4.680 |
|  |  |  |  |  | 3 | 4.296 | 4.252 |
|  |  |  |  |  | 4 | 4.379 | 4.401 |
|  |  |  |  |  | 5 | 4.625 | 4.653 |
|  |  |  |  |  | 6 | 4.392 | 4.286 |
|  |  |  |  |  | 16 | 4.141 | 4.336 |
|  |  |  |  |  | 18 | 4.290 | 4.368 |
| 14 | 3.423–4.215 | 0.258 | 3.429–4.024 | 0.187 | 1 | 4.010 | 3.909 |
|  |  |  |  |  | 2 | 4.215 | 4.024 |
|  |  |  |  |  | 3 | 3.691 | 3.633 |
|  |  |  |  |  | 4 | 3.674 | 3.701 |
|  |  |  |  |  | 5 | 3.677 | 3.683 |
|  |  |  |  |  | 6 | 3.532 | 3.423 |
|  |  |  |  |  | 16 | 3.429 | 3.581 |
|  |  |  |  |  | 18 | 3.614 | 3.687 |
| 15 | 2.753–3.499 | 0.228 | 2.723–3.354 | 0.204 | 1 | 2.829 | 2.723 |
|  |  |  |  |  | 2 | 3.499 | 3.354 |
|  |  |  |  |  | 3 | 3.008 | 2.936 |
|  |  |  |  |  | 4 | 3.124 | 3.153 |
|  |  |  |  |  | 5 | 3.014 | 3.004 |
|  |  |  |  |  | 6 | 2.903 | 2.791 |
|  |  |  |  |  | 16 | 2.753 | 2.866 |
|  |  |  |  |  | 18 | 2.983 | 3.052 |
| 16 | 0.800–1.606 | 0.277 | 0.679–1.564 | 0.288 | 1 | 1.309 | 1.197 |
|  |  |  |  |  | 2 | 1.422 | 1.410 |
|  |  |  |  |  | 3 | 1.076 | 0.962 |
|  |  |  |  |  | 4 | 1.234 | 1.273 |
|  |  |  |  |  | 5 | 1.606 | 1.564 |
|  |  |  |  |  | 6 | 0.800 | 0.679 |
|  |  |  |  |  | 16 | 0.934 | 0.940 |
|  |  |  |  |  | 18 | 0.927 | 0.980 |
| 17 | 1.313–1.992 | 0.213 | 1.306–1.944 | 0.202 | 1 | 1.709 | 1.598 |
|  |  |  |  |  | 2 | 1.992 | 1.944 |
|  |  |  |  |  | 3 | 1.413 | 1.306 |
|  |  |  |  |  | 4 | 1.487 | 1.525 |
|  |  |  |  |  | 5 | 1.524 | 1.481 |
|  |  |  |  |  | 6 | 1.464 | 1.346 |
|  |  |  |  |  | 16 | 1.443 | 1.478 |
|  |  |  |  |  | 18 | 1.313 | 1.369 |
| 18 | 1.619–2.445 | 0.252 | 1.664–2.368 | 0.224 | 1 | 2.194 | 2.085 |
|  |  |  |  |  | 2 | 2.445 | 2.368 |
|  |  |  |  |  | 3 | 1.838 | 1.741 |
|  |  |  |  |  | 4 | 1.846 | 1.882 |
|  |  |  |  |  | 5 | 1.899 | 1.864 |
|  |  |  |  |  | 6 | 1.894 | 1.778 |
|  |  |  |  |  | 16 | 1.619 | 1.664 |
|  |  |  |  |  | 18 | 1.903 | 1.963 |

**Table S5.** Mean intercept and slope before and after data screening. Each 5- point and 6-point composite standard curve’s mean intercept and slope before and after data were screened with acceptance criteria. (LB = Lower Bound; UB = Upper Bound).

| **Composite Standard Curve ID** | **Mean Intercept Before**  **(LB/UB)** | **Mean Intercept After**  **(LB/UB)** | **Mean Slope Before**  **(LB/UB)** | **Mean Slope After**  **(LB/UB)** |
| --- | --- | --- | --- | --- |
| 5-point |  |  |  |  |
| 1 | 37.887  (37.699/38.075) | 37.963  (37.756/38.171) | −3.359  (−3.416/−3.301) | −3.388  (−3.452/−3.325) |
| 2 | 38.445  (38.118/38.772) | 38.067  (37.756/38.378) | −3.532  (−3.630/−3.434) | −3.434  (−3.526/−3.341) |
| 3 | 38.075  (37.491/38.659) | 38.176  (37.773/38.579) | −3.616  (−3.793/−3.439) | −3.554  (−3.677/−3.430) |
| 4 | 38.500  (38.114/38.886) | 38.328  (37.995/38.661) | −3.480  (−3.596/−3.364) | −3.453  (−3.552/−3.353) |
| 5 | 38.332  (38.111/38.554) | 38.274  (38.053/38.495) | −3.635  (−3.700/−3.570) | −3.605  (−3.670/−3.540) |
| 6 | 38.264  (37.855/38.674) | 38.201  (38.007/38.395) | −3.659  (−3.780/−3.538) | −3.596  (−3.653/−3.539) |
| 7 | 37.889  (37.715/38.064) | 37.889  (37.715/38.064) | −3.561  (−3.612/−3.510) | −3.561  (−3.612/−3.510) |
| 8 | 38.614  (38.197/39.032) | 38.353  (38.138/38.569) | −3.630  (−3.752/−3.508) | −3.562  (−3.625/−3.499) |
| 9 | 37.803  (37.638/37.967) | 37.803  (37.638/37.967) | −3.512  (−3.560/−3.464) | −3.512  (−3.560/−3.464) |
| 10 | 38.408  (38.124/38.693) | 38.247  (38.034/38.460) | −3.712  (−3.796/−3.628) | −3.635  (−3.698/−3.572) |
| 11 | 38.163  (37.916/38.410) | 38.163  (37.916/38.410) | −3.617  (−3.690/−3.544) | −3.617  (−3.690/−3.544) |
| 12 | 37.410  (37.064/37.757) | 37.569  (37.255/37.882) | −3.518  (−3.620/−3.417) | −3.520  (−3.612/−3.429) |
| 13 | 39.428  (38.999/39.857) | 38.880  (38.613/39.146) | −3.686  (−3.812/−3.559) | −3.558  (−3.637/−3.478) |
| 14 | 37.826  (37.444/38.208) | 37.709  (37.436/37.982) | −3.452  (−3.564/−3.340) | −3.422  (−3.502/−3.342) |
| 15 | 38.782  (37.985/39.578) | 37.725  (37.373/38.076) | −3.459  (−3.693/−3.226) | −3.378  (−3.480/−3.275) |
| 16 | 38.512  (38.073/38.951) | 37.526  (37.359/37.694) | −3.621  (−3.750/−3.493) | −3.496  (−3.545/−3.447) |
| 17 | 38.350  (38.196/38.504) | 38.257  (38.103/387.412) | −3.598  (−3.645/−3.552) | −3.570  (−3.617/−3.523) |
| 18 | 37.808  (37.622/37.993) | 37.808  (37.622/37.993) | −3.546  (−3.602/−3.490) | −3.546  (−3.602/−3.490) |
| 19 | 38.322  (38.126/38.518) | 38.322  (38.126/38.518) | −3.633  (−3.692/−3.574) | −3.633  (−3.692/−3.574) |
| 20 | 38.413  (38.232/38.594) | 38.413  (38.232/38.594) | −3.557  (−3.611/−3.502) | −3.557  (−3.611/−3.502) |
| 21 | 38.315  (38.011/38.619) | 38.315  (38.011/38.619) | −3.479  (−3.571/−3.387) | −3.479  (−3.571/−3.387) |
| 6-point |  |  |  |  |
| 1 | 38.017  (37.805/38.230) | 38.017  (37.805/38.230) | −3.414  (−3.481/−3.346) | −3.414  (−3.481/−3.346) |
| 2 | NA | NA | NA | NA |
| 3 | 37.801  (37.469/38.133) | 37.873  (37.588/38.158) | −3.497  (−3.603/−3.390) | −3.464  (−3.556/−3.372) |
| 4 | 38.374  (38.092/38.657) | 38.213  (37.958/38.468) | −3.447  (−3.536/−3.357) | −3.423  (−3.503/−3.342) |
| 5 | 38.685  (38.476/38.895) | 38.527  (38.233/38.821) | −3.730  (−3.794/−3.666) | −3.667  (−3.756/−3.578) |
| 6 | 38.690  (38.383/38.998) | 38.380  (38.158/38.603) | −3.790  (−3.885/−3.696) | −3.644  (−3.712/−3.576) |
| 7 | 38.030  (37.881/38.178) | 37.947  (37.802/38.093) | −3.599  (−3.644/−3.553) | −3.576  (−3.620/−3.531) |
| 8 | 38.398  (38.117/38.679) | 38.468  (38.243/38.693) | −3.575  (−3.662/−3.488) | −3.530  (−3.599/−3.461) |
| 9 | 37.708  (37.581/37.834) | 37.708  (37.581/37.834) | −3.486  (−3.525/−3.447) | −3.486  (−3.525/−3.447) |
| 10 | 38.239  (38.004/38.473) | 38.189  (37.957/38.421) | −3.677  (−3.751/−3.604) | −3.651  (−3.724/−3.579) |
| 11 | 37.929  (37.743/38.114) | 37.929  (37.743/38.114) | −3.553  (−3.611/−3.495) | −3.553  (−3.611/−3.495) |
| 12 | 37.441  (37.190/37.693) | 37.533  (37.284/37.782) | −3.533  (−3.610/−3.456) | −3.521  (−3.597/−3.444) |
| 13 | 39.168  (38.861/39.476) | 38.728  (38.509/38.948) | −3.629  (−3.724/−3.533) | −3.528  (−3.596/−3.460) |
| 14 | 37.542  (37.308/37.776) | 37.535  (37.317/37.753) | −3.359  (−3.431/−3.286) | −3.386  (−3.454/−3.318) |
| 15 | 38.021  (37.508/38.533) | 37.856  (37.320/38.391) | −3.281  (−3.439/−3.123) | −3.491  (−3.657/−3.326) |
| 16 | 38.380  (38.059/38.702) | 37.691  (37.462/37.920) | −3.586  (−3.685/−3.487) | −3.507  (−3.577/−3.436) |
| 17 | 38.086  (37.948/38.225) | 38.086  (37.948/38.225) | −3.541  (−3.585/−3.497) | −3.541  (−3.585/−3.497) |
| 18 | 37.506  (37.334/37.678) | 37.506  (37.334/37.678) | −3.478  (−3.533/−3.424) | −3.478  (−3.533/−3.424) |
| 19 | 38.141  (37.971/38.312) | 38.141  (37.971/38.312) | −3.593  (−3.647/−3.539) | −3.593  (−3.647/−3.539) |
| 20 | 38.328  (38.180/38.476) | 38.328  (38.180/38.476) | −3.533  (−3.580/−3.486) | −3.533  (−3.580/−3.486) |
| 21 | NA | NA | NA | NA |
